# Comparison of Clinical Outcomes Between Second-and First-Generation Drug-Eluting Stents in Patients With Chronic Total Occlusion Lesion: A Meta-Analysis

**DOI:** 10.3389/fcvm.2021.598046

**Published:** 2021-04-20

**Authors:** Xuan Qiao, Wen-Jiao Zhang, Wen-Fen Guo, Yan Li, Xi-Ying Liang, Zhi-Lu Wang

**Affiliations:** ^1^The First Clinical Medical College of Lanzhou University, Lanzhou, China; ^2^Department of Cardiology, Baiyin Third People's Hospital, Baiyin, China; ^3^Department of Cardiology, The First Hospital of Lanzhou University, Lanzhou, China

**Keywords:** coronary artery disease, chronic total occlusion, meta-analysis, percutaneous coronary intervention, second-generation drug-eluting stents

## Abstract

**Background and Objectives:** The second-generation drug-eluting stents have been used to treat chronic total occlusion lesion. However, there is limited evidence of the clinical outcomes that whether the second-generation drug-eluting stents is superior to first-generation ones in patients with chronic total occlusion lesion undergoing percutaneous coronary intervention. The study aimed to compare the differences in clinical outcomes between the two generations drug-eluting stents in patients with those by a meta-analysis.

**Methods:** PubMed, Embase, the Cochrane library and Web of science databases were systemically searched before March, 2021. Randomized controlled trials and observational studies were included to compare the second-generation drug-eluting stents with the first-generation ones in patients with chronic total occlusion lesion undergoing percutaneous coronary intervention. The clinical outcomes were major adverse cardiac events (MACE), target vessel revascularization, myocardial infarction, all-cause death. Fixed effects models were used to calculate the odds ratio (OR) and 95% confidence interval (CI) of each clinical outcome. Sensitivity analysis was performed to detect potential sources of heterogeneity. Subgroup analyses were used to assess the differential effects.

**Results:** The meta-analysis included eight studies involving 4,583 patients with chronic total occlusion lesion undergoing percutaneous coronary intervention. Pooled analysis showed that the incidence of MACE (OR = 0.68, 95%CI 0.54–0.85, *P* = 0.0008), target vessel revascularization (OR = 0.70, 95%CI 0.54–0.91, *P* = 0.007), and myocardial infarction (OR = 0.58, 95%CI 0.37–0.93, *P* = 0.02) were lower in the second-generation drug-eluting stents compared with the first-generation ones. However, there was not difference in all-cause deaths between two drug-eluting stents (OR = 0.67, 95%CI 0.45–1.01, *P* = 0.05).

**Conclusions:** The second-generation drug-eluting stents are associated with lower MACE, target vessel revascularization, and myocardial infarction compared with the first-generation ones in patients with chronic total occlusion lesion undergoing percutaneous coronary intervention. The results of this study can provide a reference for the selection of stents in patients with chronic total occlusion lesion. Further randomized controlled trials are needed to verify that the second-generation drug-eluting stents is superior to the first-generation ones in patients with chronic total occlusion (Registered by PROSPERO, CRD42020158406).

## Introduction

Chronic total occlusion (CTO) lesion is characterized by the complete or near complete occlusion of coronary artery with or without minimal downstream flow (TIMI flow grade 0 or 1) for more than 3 months (1). In patients undergoing coronary angiography, CTO lesion accounted for 25%, and about 50% of CTO lesion were located in the right coronary artery (2, 3). Meanwhile, the CTO patients had a high incidence of comorbidity, such as 34% of CTO patients with diabetes, 75% with hypertension and 82% with hyperlipidemia (3). In addition, long-term CTO lesion is prone to cause myocardial ischemia and hypoxia, cardiomyopathy, leading to the decline of pump blood function, seriously affecting the health of patients (4). Infract-related artery-CTO increased the risk of ventricular tachycardia/ventricular fibrillation by more than three times (5).

Kereiakes first reported the treatment of CTO lesion with antegrade technique in 1985. Since then, percutaneous coronary intervention (PCI) has been one of the most common treatments for CTO lesion (6). With the rapid development of CTO-PCI technology and the wide application of coronary stents, the problem of in-stent restenosis is gradually exposed. The in-stent restenosis after CTO-PCI with bare mental stents was as high as 50%, which undoubtedly hindered its application in this situation (7). The first-generation drug-eluting stent (DES) was introduced to solve in-stent restenosis in 2002, and its anti-proliferative drugs can inhibit the proliferation of vascular endothelial cells and eliminate neointimal hyperplasia, the incidence of in-stent restenosis after CTO-PCI was reduced to 7–8.2% (8–10). Nevertheless, the risk of very late stent thrombosis was increased compared with bare metal stent (11). Against this background, the second-generation DES was introduced to overcome the risk of very late stent thrombosis in the first-generation DES. Compared with first-generation DES, the second-generation DES reduced the risk of very late stent thrombosis by 67–76%, and was recommended by the European Society of Cardiology guidelines (class I, level of evidence A) (12, 13). However, the evidence of the CTO-PCI used second-generation DES was limited. Randomized trials and observation studies showed no difference between the two generations stents for patients with CTO (14–19). In addition, several observation studies appear to reveal potential benefit of second-generation DES (20, 21).

Therefore, the hypothesis that the second-generation DES is superior to the first-generation DES in the treating CTO lesion was proposed. The purpose of this meta-analysis was to verify this hypothesis by comparing the differences in clinical outcomes between the two DES in patients with CTO, to seek an optimal treatment for CTO lesion and to provide evidence of clinical treatment.

## Methods

### Search Strategy and Selection Criteria

PubMed, Embase, the Cochrane library and Web of science databases were systemically searched before March, 2021. Search for the following keywords: “drug-eluting stents” AND “percutaneous coronary intervention” AND “chronic total occlusion” OR “chronic total coronary occlusion” OR “coronary chronic total occlusion” OR “CTO” without language restrictions. Set update reminder on PubMed to follow up on the latest research. The inclusion criteria for study selection were as follows: (1) patients with at least one coronary CTO; (2) comparisons of the second-generation DES (Everolimus eluting stent or Zotarolimus eluting stent or Biolimus-eluting stent) vs. the first-generation DES (Sirolimus-eluting stent or Paclitaxel-eluting stent); (3) original articles reporting at least one of these outcomes: MACE, target vessel revascularization, myocardial infarction, all-cause death, cardiac death. Exclusion criteria were: (1) duplicate publication; (2) review, conference abstract, letter, case reports; (3) study with incomplete or inaccurate data; (3) animal experiment.

### Study Identification and Quality Assessment

After removal of duplicates, the titles, abstracts and full-text articles of all articles were reviewed by two investigators (Qiao X and Zhang WJ) independently to determine the study according to the eligibility criteria. Discrepancy was solved to reduce bias through negotiation with the third party (Liang XY and Li Y). Any disagreement was resolved with third party by discussion (Wang Zhl). The quality of each randomized controlled trial (RCT) was evaluated using the Cochrane tool of Collaboration for assessing risk of bias (22). All components with low risk in a trial were considered as having a low risk of bias, trial with >one unclear risk was considered to have a moderate risk of bias and trial with >one high-risk components was considered as having a high risk of bias. The nonrandomized studies were evaluated according to the Newcastle-Ottawa scale checklist (23). This scale checklist assessed the selection, comparability and outcome of the experiment and control groups in the original study, and containing 8 items with full marks of 9 scores, 0–5 scores for low-quality literature, and 6–9 scores for high-quality literature. The present meta-analysis was performed according to the Preferred Reporting Items for Systematic Reviews and Meta-Analyses consensus statement for randomized trials and Meta-analysis of Observational Studies in Epidemiology consensus statement for non-randomized studies (24). All clinical protocols included in the study were approved by local ethics and patient informed consent. The study protocol was registered in PROSPERO (CRD42020158406).

### Data Acquisition and Clinical Outcomes

Data on participants and procedural characteristics, follow-up duration, and the clinical outcomes were extracted independently by two researchers (Qiao X and Zhang WJ) from the original publications, and negotiate divergences with the two independent authors (Liang XY and Li Y). All data extracted were checked by the last author (Wang Zhl). The clinical outcomes were MACE, target vessel revascularization, myocardial infarction, all-cause death. The MACE was defined as the original report. The target vessel revascularization was defined as any repetitive revascularization of the target vessel. The all-cause death was defined as death attributed to various causes.

### Statistical Analysis

Outcomes were analyzed by an intention-treat analysis. Continuous variables were expressed as averages or medians, and categorical variables were described using absolute numbers and proportions (%). Statistical software such as Review Manager Version 5.3 software (The Nordic Cochrane Centre, Copenhagen, Denmark) and Stata version 12.0 software (Statacorp LP, College Station, Texas, USA) were used for statistical analysis. The Mantel-Haenszel method was applied to calculate the odds ratio (OR) and 95% confidence interval (CI) of each outcome. Heterogeneity was assessed by the Cochrane Q statistic with Pearson chi-square test and the Higgins *I*^2^ test. In case of less heterogeneity (*I*^2^ < 50%, fixed effect model was utilized, otherwise random effect model was performed for calculate the pooled OR. In addition, sensitivity analysis was performed to determine whether the omittance of a single study result affected the stability of the overall results. Subgroup analysis was utilized to assess the differential effects. Two-tailed *P-*values were exploited for all results, and statistical significance set at *P* < 0.05. Visual estimation of funnel plot and the Begg's and Egger's tests was used to detect the possibility of publication bias.

### Trial Sequential Analysis

Trial Sequential Analysis version 0.9.5.10 software (Copenhagen Trial Unit, CTU) was used to evaluate the random errors and the required information size of clinical outcomes, which based on an α of 0.05 and a power of 0.8 for RCTs.

## Results

### Search Results and Study Characteristics

**Figure 1:**
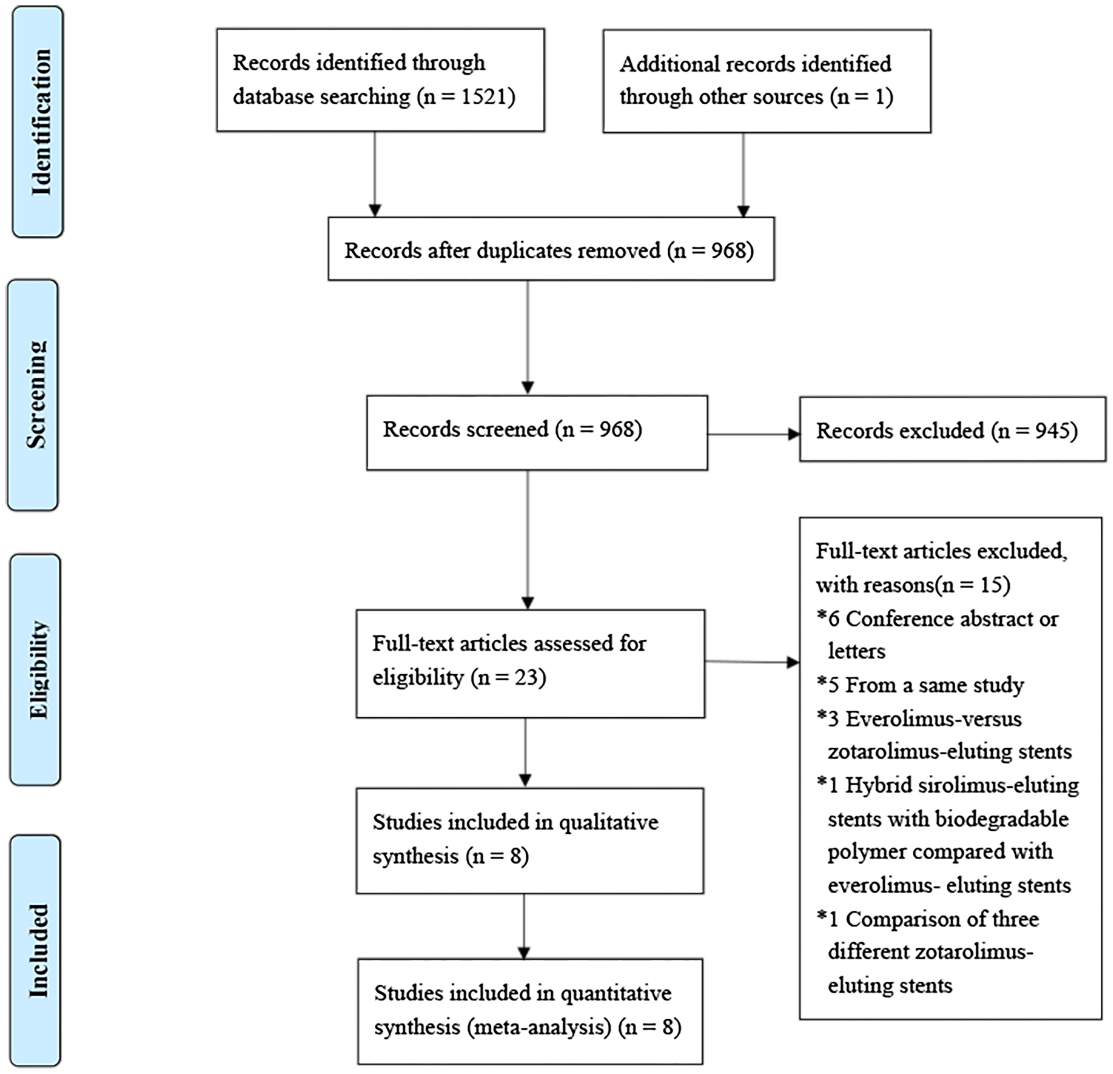
Flow diagram of literature search.

The search results are shown (Figure 1). The initial search retrieved 1,522 articles from medical databases and other sources, of which 554 were duplicates. After browsing titles, abstract and reading the full text, two randomized controlled trials and six nonrandomized controlled studies containing 4,583 patients with CTO lesion undergoing PCI were determined. Among them, three studies included 637 patients of Caucasian and five studies contained 3,946 patients of Asian, 1,801 (39%) patients used second-generation DES and 2,782 (61%) received first-generation DES. The baseline characteristics of the studies included are presented (Table 1). The duration of follow-up in the study included was from 9 months to 60 months. The majority of participants were males, which accounted for 78%, 61% of patients had multi-vessel diseases. The average age of the patients used the second -generation DES was from 58 to 60 years, while that of patients undergoing the first -generation DES was from 61 to 68 years. CTO patients with hypertension accounted for 63%, diabetes 35% and hyperlipidemia 32% in this studies. The mean LVEF of patients was from 43 to 58%. The baseline characteristics and procedure of the participants included are summarized (Table 2).

**Table 1:** Baseline characteristics of the studies included.

**Trails**	**Region**	**RCT**	**Enrolment years**	***N***	**Definition of CTO**	**The characteristics of patients**	**Stent type**	**MACE**	**Follow-up time** ** (months)**
Valenti et al. (20)	Italy	No	2006–2011	258	Patients with CTO with an estimated duration of >3 months	Any CAD and patients with long occlusions, extensive calcification, bridging collaterals, a nontapered stump, or a side branch at the occlusion site	EES/PES	Cardiovascular death + MI + TVR	9
Park et al. (14)	Koera	Yes	2007–2010	160	Patients with CTO with an estimated duration ≥1 month	Any CAD	ZES/SES	–	12
Moreno et al. (15)	Spain and Portugal	Yes	2008–2010	207	Patients with CTO with an estimated time since occlusion of >2 weeks	Angina, silent ischemia	EES/SES	Death + MI + TVR	12
Jaguszewski et al. (21)	Poland	No	2006–2011	172	Patients with CTO with and estimated time of coronary occlusion of at least 3 months.	Angina and highly selected setting of isolated CTO	ZES EES BES/PES SES	One or more of the following: all-cause death, non-fatal MI, urgent, and emergent revascularization	24
Lee et al. (19)	Koera	No	2007–2009	1,509	Patients with CTO an estimated duration 3 months	Any CAD	EES/PES SES	Composite of CD, non-fatal MI, and TLR	12
Ahn et al. (16)	Koera	No	2003–2012	1,006	Patients with CTO with an estimated duration longer than 3 months	Any CAD	ZES/PES SES	Cardiac death + MI + repeat revascularization (included TVR-PCI, non–TVR-PCI, or CABG).	24
Cho et al. (17)	Korea	No	2003–2015	1,049	Patients with CTO with an estimated duration of more than 3 months	Any CAD	ZES EES BES/PES SES	Composite of death, Q-wave MI or TVR	36
Kim et al. (18)	Korea	No	2004 −2015	222	Patients with CTO at least 3 months	Any CAD	ZES EES/PES SES	All-cause death, recurrent-MI, total repeat revascularization (TLR, TVR, and non-TVR)	60

*RCT, randomized controlled trial; CTO, chronic total occlusion; MACE, major adverse cardiac events; CAD, coronary artery disease; ZES, zotarolimus-eluting stent; EES, everolimus-eluting stent; BES, biolimus -eluting stent; SES, sirolimus-eluting stent; PES, paclitaxel -eluting stent; MI, myocardial infarction; TVR, target vessel revascularization; CD, cardiac death; TLR, target lesion revascularization; PCI, percutaneous coronary intervention; CABG, coronary artery bypass graft*.

**Table 2:** Baseline characteristics of the included trials.

	**Randomized controlled trial**									**Nonrandomized trials**							
**Study**	**Park et al**. **(*****N*** **=** **160)**		**Moreno et al**. **(*****N*** **=** **207)**		**Valenti et al**. **(*****N*** **=** **258)**		**Jaguszewski et al**. **(*****N*** **=** **172)**		**Lee et al**. **(*****N*** **=** **1,509)**			**Ahn et al**. **(*****N*** **=** **1,006)**		**Cho et al**. **(*****N*** **=** **1,049)**		**Kim et al**. **(*****N*** **=** **222)**	
**Year**	**2011**		**2013**		**2011**		**2014**		**2015**			**2016**		**2016**		**2019**	
**Variables**	**2nd gen-DES**	**1st gen-DES**	**2nd gen-DES**	**1st gen-DES**	**2nd gen-DES**	**1st gen-DES**	**2ND gen-DES**	**1st gen-DES**	**2nd gen-DES**	**1st gen-DES**		**2nd gen-DES**	**1st gen-DES**	**2nd gen-DES**	**1st gen-DES**	**2nd gen-DES**	**1st gen-DES**
Stent type	ZES	EES	EES	SES	EES	PES	ZES/EES/BES	SES/PES	EES	PES	SES	ZES/EES	SES/PES	EES/ZES/BES	SES/PES	ZES/EES	SES/PES
Participants (*n*)	80	80	106	101	112	146	23/38/9	37/65	311	556	642	168/280	331/226	311/226/25	396/91	88/23	37/74
Age (years)	62.7	63	65	63	68	67.4	63.3	63.8	62.3	62.3	61.6	62.8	62.3	60	58.8	61.6	61.1
Male (%)	52 (65)	61 (76)	85 (80)	87 (86)	98 (87)	130 (89)	56 (80)	72 (70.6)	236 (75.9)	414 (74.5)	465 (72.4)	336 (74.8)	448 (80.4)	471 (83.8)	394 (80.9)	83 (74.8)	86 (77.5)
Hypertension (%)	52 (65)	50 (63)	73 (69)	68 (67)	69 (62)	83 (57)	53 (75.7)	76 (74.5)	208 (67.1)	348 (63.3)	407 (63.7)	288 (64.1)	338 (60.7)	355 (63.2)	275 (56.5)	59 (62.2)	71 (64.0)
Diabetes (%)	28 (35)	23 (29)	43 (41)	32(32)	29 (26)	38 (26)	26 (37.1)	36 (35.3)	126 (40.6)	202 (36.6)	213 (33.3)	177 (39.4)	231 (41.5)	173 (30.8)	145 (29.8)	40 (36.0)	41 (36.9)
Hyperlipidemia (%)	21 (26)	15 (19)	70 (66)	78 (77)	62 (50)	55 (51)	43 (61.4)	57 (55.9)	124 (40.5)	202 (36.6)	241 (38.6)	221 (49.2)	231 (41.5)	–	–	30 (27.0)	27 (24.3)
Current smoker (%)	28 (35)	30 (38)	53 (50)	68 (67)	17 (15)	32 (22)	13 (18.6)	15 (14.7)	124 (40.5)	202 (36.6)	241 (38.6)	142 (31.6)	171 (30.7)	163 (29.0)	108 (22.2)	40 (36.0)	50 (45.0)
Previous MI (%)	9 (11)	8 (10)	35 (33)	43 (42.6)	71 (63)	81 (55)	50 (71.4)	60 (58.8)	37 (12.0)	81 (14.6)	82 (12.9)	–	–	56 (10.0)	47 (9.6)	11 (9.9)	11 (9.9)
Previous PCI (%)	11 (14)	15 (19)	27 (25.5)	43 (42.6)	45 (40)	47 (32)	51 (72.8)	68 (66.7)	61 (19.6)	130 (23.5)	149 (23.3)	91 (20.3)	119 (21.4)	146 (26.0)	119 (24.4)	17 (15.3)	25 (22.5)
Previous CABG (%)	–	–	5 (4.7)	4 (4)	13 (12)	19 (13)	–	–	8 (2.6)	16 (2.9)	12 (1.9)	–	–	–	–	–	–
LVEF (%)	57	57	52	54	45	43	45	50	–	–	–	57.4	57.5	57.7	58	51.6	50.9
ACS (%)	–	–	29 (27)	35 (34)	30 (27)	52 (36)	–	–	107 (57)	199 (54)	288 (55)	149 (33)	150 (27)	121 (21.5)	148 (30.4)	22 (19.8)	20 (18)
Stable CAD (%)	62 (78)	60 (75)	77 (73)	67 (66)	–	–	69 (99)	101 (99)	82 (43)	168 (46)	238 (45)	–	–	441 (78.5)	339 (69.6)	–	–
**Treated vessels**																	
LM (%)	–	–	1 (1)	0 (0)	–	–	–		2 (0.7)	3 (0.5)	4 (0.6)	3(0.7)	0(0)	0 (0)	2 (0.4)	–	–
LAD (%)	31 (39)	44 (55)	43 (40.6)	42 (41.6)	39 (35)	46 (31)	20 (28.6)	27 (26.5)	112 (40.9)	205 (37.0)	334 (52.2)	170 (37.9)	243 (43.6)	249 (43.3)	228 (46.4)	49 (44.1)	41 (36.9)
LCX (%)	15 (19)	15 (19)	18 (17)	21 (20.8)	15 (13)	20 (14)	27 (38.6)	22 (21.6)	55 (20.1)	116 (20.9)	108 (16.8)	145(32.3)	149(26.8)	71 (12.3)	79 (16.1)	27 (24.3)	35 (31.5)
RCA (%)	34 (42)	21 (26)	45 (42.5)	36 (35.6)	58 (52)	80 (55)	23 (32.8)	53 (51.9)	105 (38.3)	230 (41.5)	196 (30.5)	185 (41.2)	234 (42)	254 (44.2)	181 (36.9)	42 (37.8)	44 (39.6)
Multivessel disease (%)	54 (68)	58 (72)	56 (55.4)	60 (56.6)	94 (84)	124 (85)	34 (49)	41 (40)	206 (66)	408 (64)	373 (67)	311 (69.3)	394 (70.7)	204 (54.1)	268 (55.0)	54 (48.6)	56 (50.5)
IVUS (%)	35 (44)	40 (50)	–	–	–	–	–		–	–	–	–	–	540 (93.9)	399 (81.3)	–	–
Total stent length (mm)	43.4	44.6	49.8	47.5	72	69	33.15	32.7	40.8	42.9	40.7	31.9	35	–	–	40.5	40.8

*2nd gen-DES, the second- generation DES; 1st gen -DES, the first- generation DES; ZES, zotarolimus-eluting stent; EES, everolimus-eluting stent; BES, biolimus -eluting stent; SES, sirolimus-eluting stent; PES, paclitaxel -eluting stent; MI, myocardial infarction; PCI, percutaneous coronary intervention; CABG, coronary artery bypass graft; LVEF, left ventricular ejection fraction; ACS, acute coronary syndrome; CAD, coronary artery disease; LM, left main; LAD, left anterior descending; LCX, left circumflex; RCA, right coronary artery; IVUS, intravascular ultrasound*.

### Quality Assessment and Risk of Bias

The Cochrane quality assessment shows that there was one unclear risk of bias in two randomized trials, which had a moderate risk of bias (Supplementary Table 1). The quality assessment of nonrandomized studies evaluated by the Newcastle-Ottawa scale checklist present that five studies had scores ≥6 points, which were high quality literature, and one study had scores < 6 points, which were low quality literature (Supplementary Table 2).

### Trial Sequential Analysis

Since one of two RCTs did not have data on the clinical outcomes of MACE or all-cause death, only target vessel revascularization and myocardial infarction were assessed for random errors and the required information size. The cumulative Z-curve does not cross either the conventional boundary or the trial sequential monitoring boundary, which suggested there was no significant difference between the second-generation DES and the first-generation DES in treatment of target vessel revascularization and myocardial infarction (Supplementary Figure 1). The trial sequential analysis of these two RCTs presented that the comparison of the second-generation DES and the first-generation DES in the treatment of target vessel revascularization and myocardial infarction were far from sufficient in terms of the required information size, and further RCTs are needed to determine whether the second-generation DES is superior to the first-generation DES in the treatment of target vessel revascularization and myocardial infarction.

### Clinical Outcomes

**Figure 2:**
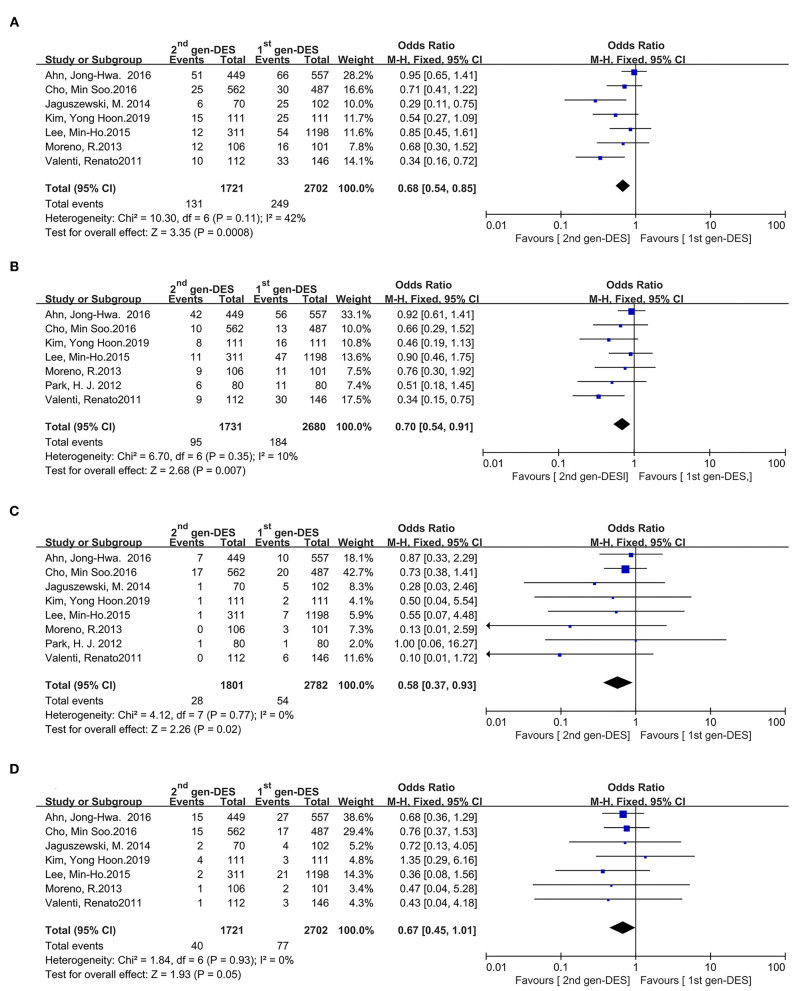
Comparison of clinical outcomes between the second- and first-generation DES groups. **(A)** major adverse cardiac events, **(B)** target vessel revascularization, **(C)** myocardial infarction, **(D)** all-cause death. 2nd gen-DES, the second- generation DES; 1st gen -DES, the first- generation DES.

The MACE is appeared in seven studies, the second-generation DES provided a significant advantage over the first-generation DES in reducing the incidence of MACE in Figure 2A (OR = 0.68, 95%CI 0.54–0.85, *P* = 0.0008), and without significant heterogeneity among studies (*I*^2^ = 42%, *P*_*heterogeneity*_ = 0.11). According to visual estimation, the funnel plot was asymmetrical and publication bias was found in Egger's and Begg's tests (*P* = 0.042 and *P* = 0.019, respectively) (Supplementary Figure 2A). Similarly, Seven of the eight studies had a data on the target vessel revascularization, whose rate are significantly reduced in patients with second-generation DES compared with the patients with first-generation DES in Figure 2B (OR = 0.70, 95% CI 0.54–0.91, *P* = 0.007), there was no significant heterogeneity across the enrolled trials (*I*^2^ = 10%, *P*_*heterogeneity*_ = 0.35). The funnel plot showed slightly asymmetrical but Egger's and Begg's tests did not show publication bias (*P* = 0.368 and *P* = 0.089, respectively) (Supplementary Figure 2B). Meanwhile, compared with the first-generation DES group, the second-generation DES group has a trend to reduce the incidence of myocardial infarction in all eight studies in Figure 2C (OR = 0.58, 95% CI 0.37–0.93, *P* = 0.02), the analysis revealed no significant heterogeneity among studies (*I*^2^ = 0%, *P*_*heterogeneity*_ = 0.77). Although the funnel plot presented asymmetry according to visual estimation, there was no publication bias according to Egger's and Begg's tests (*P* = 0.063 and *P* = 0.053, respectively) (Supplementary Figure 2C). Conversely, seven of the eight studies show that the all-cause death of the second-generation DES was not significantly lower than that of the first-generation DES in Figure 2D (OR = 0.67, 95% CI 0.45–1.01, *P* = 0.05) and there was no significant heterogeneity among studies (*I*^2^ = 0%, *P*_*heterogeneity*_ = 0.93). The funnel plot exhibited symmetry and there was no publication bias in Egger's and Begg's tests (*P* = 0.764 and *P* = 0.560, respectively) (Supplementary Figure 2D).

### Subgroup and Sensitivity Analysis

Since the total length of stent implanted in most studies was ≥ 40 mm, the 40 mm was used as the cut-off point of stent length for subgroup analysis, and subjects were CTO patients with long lesions and multiple stents implanted (20). Meanwhile, the race (Asian vs. Caucasian) and study designs (RCT vs. Non-RCT) were analyzed as subgroup. The occurrence of MACE, target vessel revascularization and myocardial infarction in CTO patients with second-generation DES are lower than that in those patients with first-generation DES when the total length of stent implanted ≥ 40 mm (Supplementary Figures 3A–C). Similarity, when the study designs of non-RCT and the race of Caucasian, the second-generation DES perform better than the first-generation DES in incidence of MACE, target vessel revascularization and myocardial infarction in CTO patients (Supplementary Figures 3A–C). However, the incidence of all-cause death seems to be no significant difference caused by the study design, race and total length of stent implanted (Supplementary Figure 3D). In addition, the original study analyzed clinical outcome by intention-treat analysis and no separate data were available, subgroup analysis also did not apply to differences in stent types.

There is a moderate statistical heterogeneity in the MACE (*I*^2^ = 42%) (Figure 2A). Sensitivity analysis revealed that the study by Ahn, Jong-Hwa in 2016 is the source of statistical heterogeneity for the MACE in the meta-analysis (Supplementary Figure 4) (16). After excluding the results of this study, the statistical significance of the MACE pooled analysis remains unchanged (OR = 0.57, 95%CI 0.43–0.76, *P* < 0.0001, while heterogeneity among the remaining studies is reduced (*I*^2^ = 19%) (Supplementary Figure 5).

## Discussion

This meta-analysis enrolled two RCTs and six nonrandomized controlled studies, which compared the clinical outcomes associated with the second-generation DES and the first-generation DES in patients with CTO lesion undergoing PCI. The principal finding is that the second-generation DES was superior to reduce the incidence of MACE, target vessel revascularization and myocardial infarction in patients with CTO lesion compared with the first-generation DES. In contrast, the use of the second-generation DES did not appear a lower incidence of all-cause death than that of the first-generation DES.

In 1985, the success rate of antegrade wire technique in treating CTO lesion was only 53% (4). In recent years, with the application of antegrade wire technique, retrograde wire technique, antegrade dissection re-entry and hybrid strategy, success rates of CTO-PCI have significantly increased while maintaining low risk of complications (25). The first-generation DES significantly reduced the rates of restenosis in the treatment of CTO lesion compared with bare-metal stent, but the stent coated with permanent materials increased the risk of very late stent thrombosis, which defined as more than 360 days after PCI according to the Academic Research Consortium (11, 26). Although the incidence of very late thrombosis after PCI is as low as 0.4–2% under dual antiplatelet therapy, the occurrence of stent thrombosis is devastating, usually presenting as major myocardial infarction that often requires reintervention (27, 28). The second-generation DES as a novel therapeutic material, which have thinner scaffolds and more biocompatible polymers, seems to demonstrate potential benefits, which were widely used to improve safety, reduce the dose of antiproliferative drugs and ameliorate the release kinetics. The EXPERT CTO Multicenter trial published in 2015 showed that it is beneficial to use the everolimus-eluting stent in patients with CTO lesion (29). However, the use of the first-generation DES and the second-generation DES is still controversial. In particular, whether the second-generation DES is better than the first-generation DES in the treatment of CTO lesion, there is no basis for guidance and consensus. Therefore, it is necessary to explore the clinical outcome of two generations of stent in the treatment of CTO lesion.

This meta-analysis showed that the second-generation DES had a low incidence tendency of MACE, target vessel revascularization, and myocardial infarction compared with the first-generation one. However, there was no difference in all-cause deaths between the two generation DES, which was similar to the results of a meta-analysis by Lanka et al. in 2014 (30). That meta-analysis enrolling 1,174 patients showed that the second-generation DES were associated with a lower incidence of death, target vessel revascularization compared with the first-generation DES, but the incidence of myocardial infarction and stent thrombosis was similar (30), while most participants in this study had multi-vessel diseases (61%) and the total length of stent implantation was more than 40 mm. Further analysis showed that the second-generation DES was better than the first-generation DES in these patients with multi-vessel lesion, long occlusive lesion requiring multiple stent implantation (total stent length ≥ 40 mm). Similarly, the incidence of MACE, target vessel revascularization and myocardial infarction in Caucasian in the second-generation DES might be lower than that in the first-generation DES. It suggests that patients with CTO lesion in Caucasians are more suitable for the second- generation DES than those in Asians.

In the initial pooled analysis, the second-generation DES group was better than the first-generation DES group in reducing the incidence of MACE. However, there was moderate heterogeneity. Further sensitivity analysis showed that one study with clinical heterogeneity was the source of statistical heterogeneity. This heterogeneity may be due to the fact that the total length of stent implantation was < 40 mm in most of Asian population analyzed in this study. The results showed that there was no significant difference between the second- and first-generation DES (HR = 1.00, 95%CI 0.67–1.50, *P* = 0.99). Therefore, it is considered that this study has an obvious clinical heterogeneity, which is the main source of MACE pooled analysis heterogeneity. It is worth noting that the results of the subgroup analysis showed that the second-generation DES was better than the first-generation DES when the total length of stent implantation ≥ 40 mm and in Caucasian. Combined with sensitivity analysis and subgroup analysis, the results demonstrate that the incidence of MACE tends to be reduced in the second-generation DES group. In addition, the second-generation DES in the non-RCT subgroup had a lower incidence of MACE, target vascular revascularization and myocardial infarction (consistent with the results of the initial pooled analysis) in the subgroup analysis, while there was no difference between the two generations DES in the RCT subgroup. Combined with this subgroup analysis and the trial sequential analysis evaluation of the RCTs, the heterogeneity may be due to intergroup one between RCT and non-RCT (differences of study design). On the one hand, although 3 months was recognized as the accepted cutoff, different studies had definitions of CTO lesion. In observational studies, the estimated occlusion time of CTO lesion was >3 months. In the RCTs of CATOS, the estimated occlusion time of CTO lesion was ≥1 month, while the estimated occlusion time of lesion was ≥2 weeks in the CIBELES trial (14, 15), which may affect the results by including partial patients with subtotal occlusion. On the other hand, clinical heterogeneity among patients included in observational studies may also be an important source of heterogeneity. The study by Valenti showed that the second-generation DES was significantly superior to the first-generation DES in reducing the incidence of MACE (22.6 vs. 8.9%, *P* = 0.003), target vessel revascularization (20.5 vs. 8%, *P* = 0.005) and in-stent restenosis or re-occlusion (31.4 vs. 11.8%, *P* = 0.001) in patients with long occlusions, extensive calcification, bridging collaterals and non-tapered stump (20). The study by Jaguszewsk enrolled highly selected patients with isolated CTO, and which showed that compared with first-generation DES, the second-generation DES significantly reduced the risk of MACE during 1 year follow-up (2.8 vs. 17.6%, HR = 0.15, 95%CI, 0.06–0.36, *P* = 0.01) (21). It is suggested that the second-generation DES is superior to the first-generation DES, indicating that the second-generation DES has excellent performance in complex CTO lesion. In conclusion, it is still believed that the second-generation DES has a significant advantage over the first-generation DES in reducing the incidence of MACE, target vascular revascularization and myocardial infarction. Other clinical outcome such as all-cause death did not show significant heterogeneity.

Although this result shows that the second-generation DES can reduce the incidence of MACE, target vessel revascularization and myocardial infarction in patients with CTO compared with the first-generation DES. However, the interpretation of this result should be cautious. First of all, since most of the patients in this study are male, it is not clear whether there are gender differences in the results of this study. Therefore, whether the second-generation DES has clinical efficacy compared with the first-generation DES needs to be further confirmed for female patients with CTO. Secondly, most of the patients in this study have been implanted the second-generation DES, while the number of patients implanted the first-generation DES is very small, which may affect this result. Thirdly, race and patients with multi-vessel lesion, long occlusive lesion requiring multiple stents should be considered, the Asian population and isolated CTO patients need to weigh the advantages and disadvantages to make a prudent decision. Fourthly, trial sequential analysis evaluations show that the sample size of two RCTs has not reached the expected information of trial sequential analysis. The pooled results are driven by observational studies. Therefore, clinicians need to make prudent decisions regarding non-highly selective individual CTO patients, such as CTO patients with acute coronary syndromes or bifurcation lesion. Finally, the success rate of coronary interventional in the treatment of CTO mainly depends on the operation of the guide wire, the characteristics of the lesion and the situation of the patients. These factors also need to be considered.

## Limitation

The limitations of this study should be recognized. First, although the combination of MeSH and free-words is adopted in literature retrieval and manual retrieval in this study, it is still possible to ignore the original research and affect the analysis results. Second, the data of this study are mainly from observational studies, including only two randomized studies (14, 15), which cannot exclude differences in baseline characteristics, methodology, drug therapies. In the absence of randomized observational studies, measured or unmeasured confounding factor may affect the pooled results. Third, the difference of the race populations may be an important source of heterogeneity. The second-generation DES was beneficial to Caucasian patients from three studies (15, 20, 21), which supports the applicability of this result. Fourth, despite the effects of multi-vessel lesion and long occlusive lesion. However, the subgroup analysis showed that patients with total length of stent implantation more than 40 mm may be suitable for the second-generation DES. Fifth, the visual estimation of the funnel plot and Egger's and Begg's tests detected publication bias of the MACE, which may be due to the fact that fewer than 10 studies were included and the results may be conservative. Final, the use of various crossing strategies and treated different vessels may be an important source of heterogeneity.

## Conclusion

This meta-analysis supports that the use of second-generation DES is superior to first-generation DES in the treatment of CTO lesion, and the former has significant clinical outcomes in reducing MACE, target vessel revascularization, myocardial infarction in patients with CTO. Further large-scale, well-designed RCTs of patients with CTO are also needed to assess the benefits of the second-generation DES in certain subgroups, such as highly selective individual CTO patients or patients with Asian population, or patients using the same crossing strategies.

## Data Availability Statement

The original contributions presented in the study are included in the article/Supplementary Material, further inquiries can be directed to the corresponding author/s.

## Author Contributions

XQ: writing - original draft, methodology, and software. W-JZ: formal analysis, visualization, and software. W-FG and X-YL: validation and data curation. YL: investigation and validation. Z-LW: conceptualization and supervision. All authors contributed to the article and approved the submitted version.

## Conflict of Interest

The authors declare that the research was conducted in the absence of any commercial or financial relationships that could be construed as a potential conflict of interest.
