# Supplementary material for: Comparison of Clinical Outcomes Between Second-and First-Generation Drug-Eluting Stents in Patients With Chronic Total Occlusion Lesion: A Meta-Analysis
Source: Front Cardiovasc Med. 2021 Apr 20;8:598046. doi: 10.3389/fcvm.2021.598046 (PMC8096061; doi:10.3389/fcvm.2021.598046)
Supplement: Supplementary file 1 [file Data_Sheet_1.docx]

**SUPPLEMENTARY DATA**

**Comparison of clinical outcomes between second-and first- generation drug-eluting stents in patients with chronic total occlusion lesion: a meta-analysis**

**List of Supporting Information Content**

**Supplementary Fig.1.** Trial Sequential Analysis of RCTs for clinical outcomes of (A) target vessel revascularization, (B) myocardial infarction.

Notes: RIS, required information size.

**Supplementary Fig.2.** Funnel plot for each clinical outcome of (A) major adverse cardiac events, (B) target vessel revascularization, (C) myocardial infarction, (D) all-cause death.

**Supplementary Fig.3.** Subgroup analysis for clinical outcomes of (A) major adverse cardiac events, (B) target vessel revascularization, (C) myocardial infarction, (D) all-cause death.

**Supplementary Fig. 4.** Sensitivity analysis of MACE.

**Supplementary Fig.5.** Comparison of MACE between the second- and first-generation DES groups after excluding one study.

Notes: 2^nd^ gen-DES, the second- generation DES; 1^st^ gen -DES, the first- generation DES.

**Supplemental Table 1.** Risk assessment of bias for randomized controlled trials included.

**Supplemental Table 2**. Risk assessment of bias for observation studies included.
