# Supplementary material for: Comparison of Clinical Outcomes Between Second-and First-Generation Drug-Eluting Stents in Patients With Chronic Total Occlusion Lesion: A Meta-Analysis
Source: Front Cardiovasc Med. 2021 Apr 20;8:598046. doi: 10.3389/fcvm.2021.598046 (PMC8096061; doi:10.3389/fcvm.2021.598046)
Supplement: Supplementary file 2 [file Data_Sheet_2.docx]

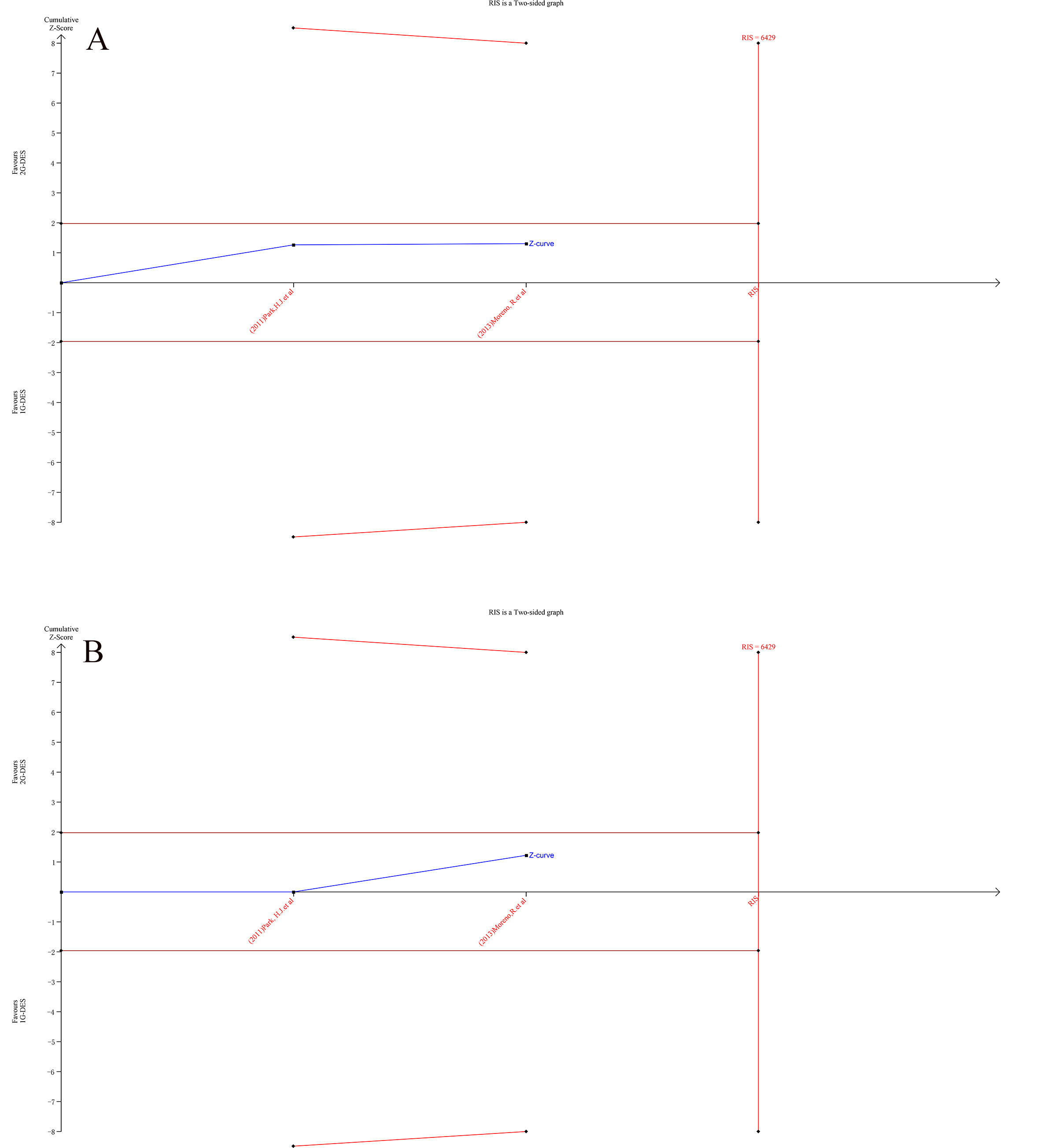


Supplementary Fig.1. Trial Sequential Analysis of RCTs for clinical outcomes of (A) target vessel revascularization, (B) myocardial infarction.

Notes: RIS, required information size.





Supplementary Fig.2. Funnel plot for each clinical outcome of (A) major adverse cardiac events, (B) target vessel revascularization, (C) myocardial infarction, (D) all-cause death.


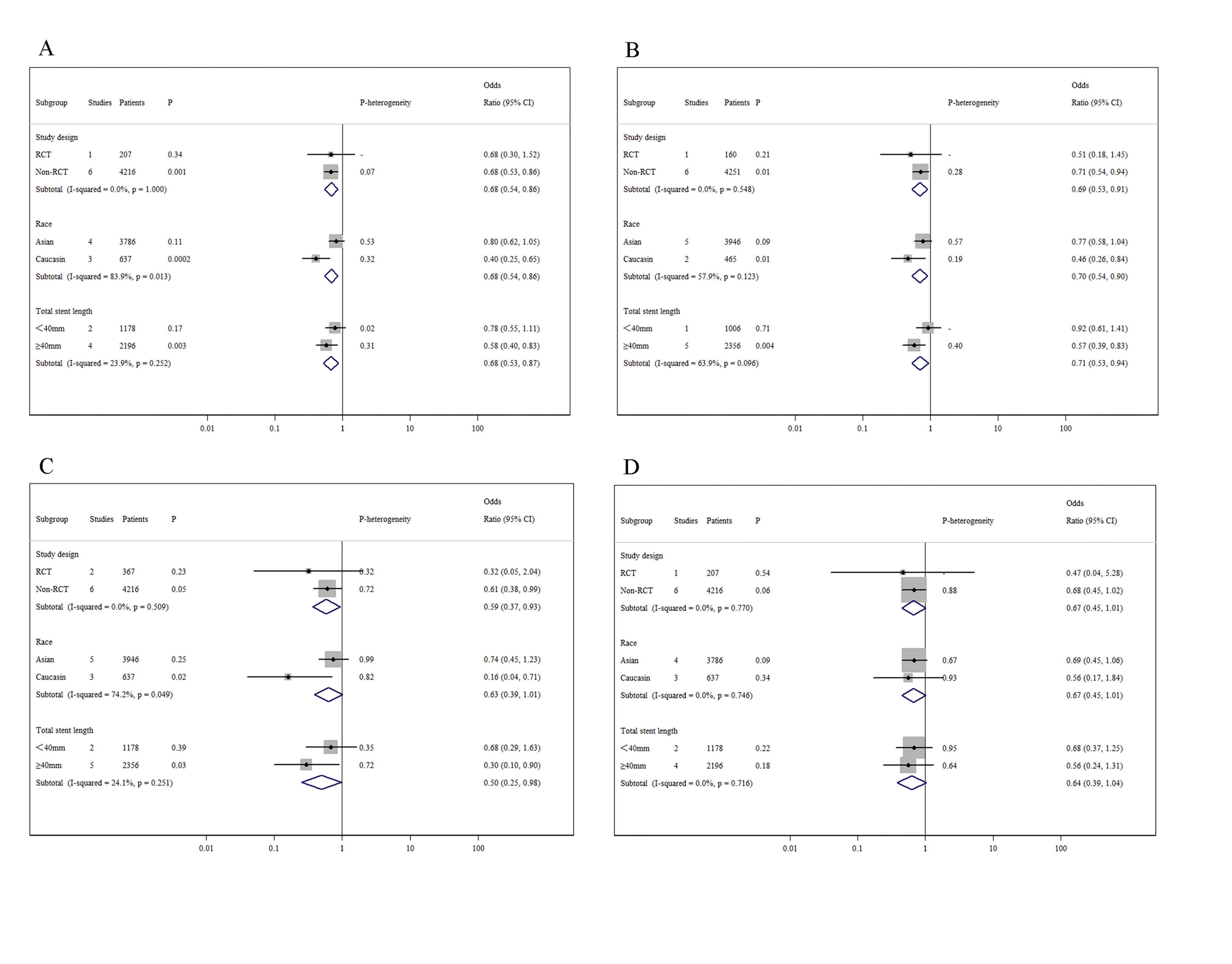


Supplementary Fig.3. Subgroup analysis for clinical outcomes of (A) major adverse cardiac events, (B) target vessel revascularization, (C) myocardial infarction, (D) all-cause death.


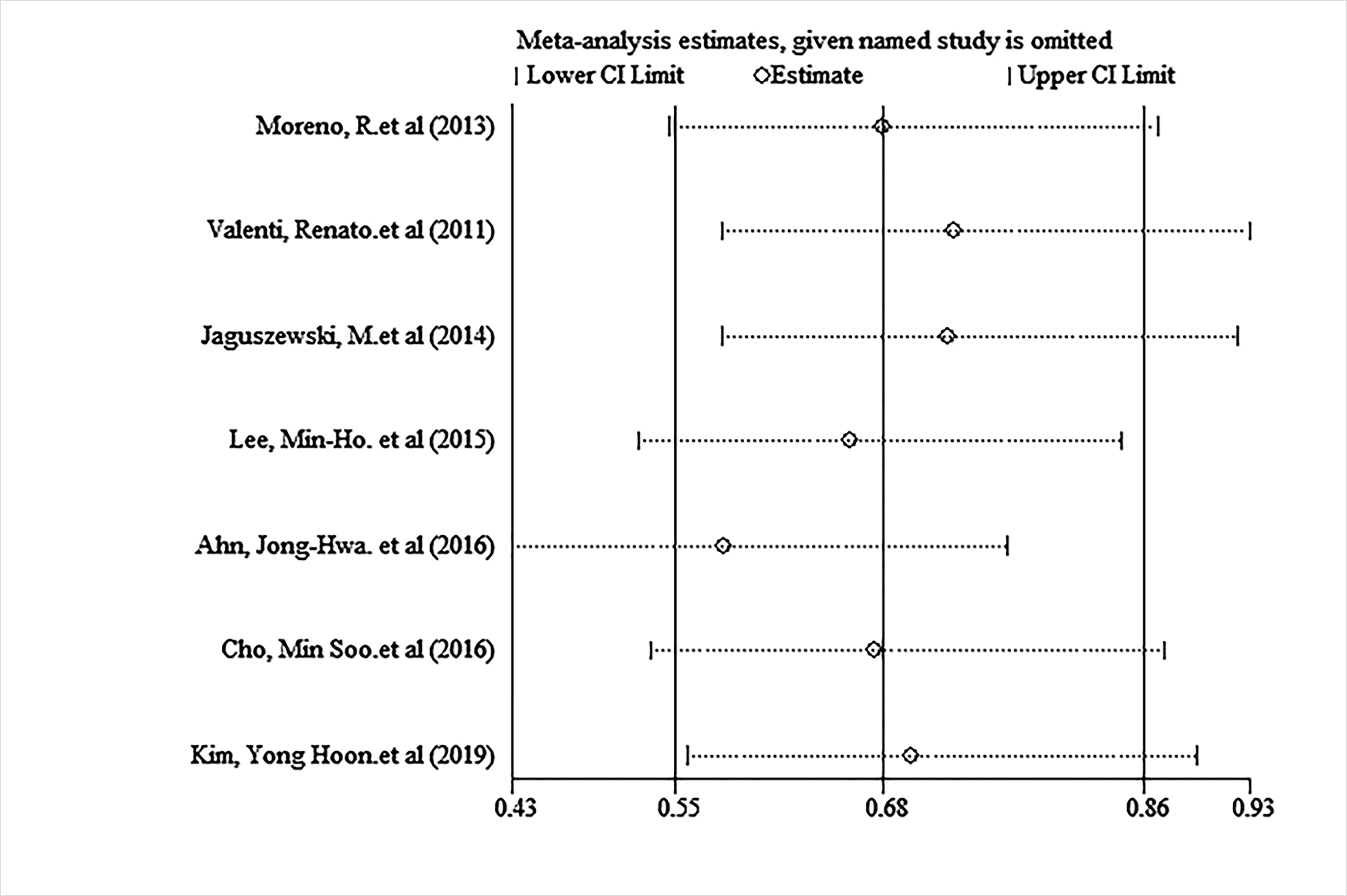


Supplementary Fig. 4. Sensitivity analysis of MACE.


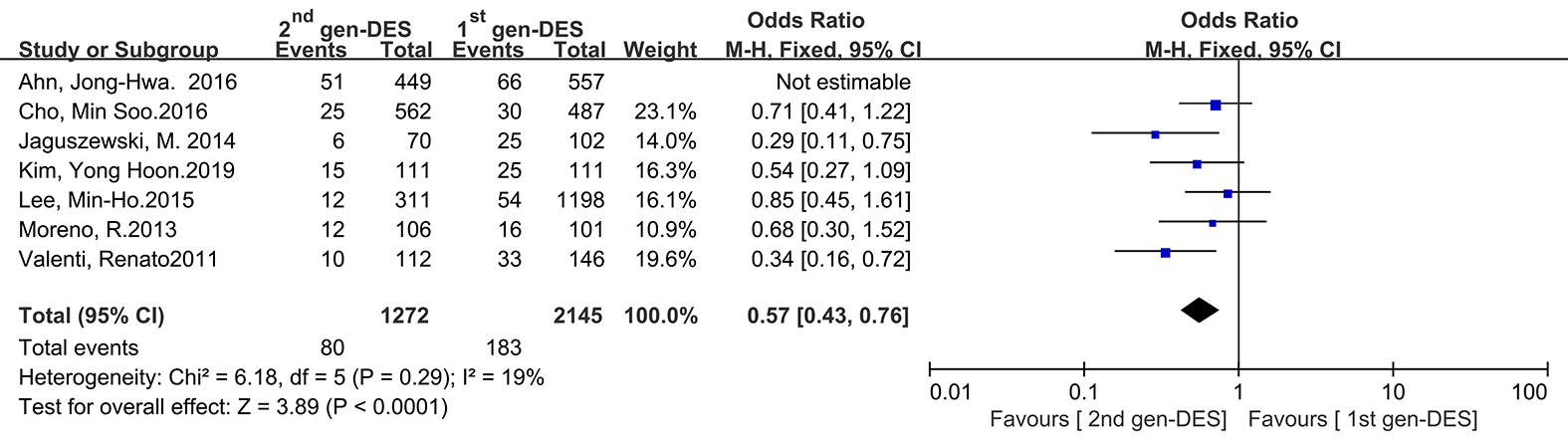


Supplementary Fig.5. Comparison of MACE between the second- and first-generation DES groups after excluding one study.

Notes: 2^nd^ gen-DES, the second- generation DES; 1^st^ gen -DES, the first- generation DES.
